# Specific white matter tissue microstructure changes associated with obesity

**DOI:** 10.1016/j.neuroimage.2015.10.006

**Published:** 2016-01-15

**Authors:** Stephanie Kullmann, Martina F. Callaghan, Martin Heni, Nikolaus Weiskopf, Klaus Scheffler, Hans-Ulrich Häring, Andreas Fritsche, Ralf Veit, Hubert Preissl

**Affiliations:** a Institute for Diabetes Research and Metabolic Diseases of the Helmholtz Center Munich at the University of Tübingen, Tübingen, Germany; b German Center for Diabetes Research, Tübingen, Germany; c Institute of Medical Psychology and Behavioral Neurobiology, University of Tübingen, Germany; d Wellcome Trust Centre for Neuroimaging, UCL Institute of Neurology, University College London, 12 Queen Square, WC1N 3BG London, UK; e Department of Internal Medicine IV, University of Tübingen, Germany; f Department of Neurophysics, Max Planck Institute for Human Cognitive and Brain Sciences, Leipzig, Germany; g Department of Biomedical Magnetic Resonance, University of Tübingen, Germany; h Department of High-Field Magnetic Resonance, Max Planck Institute for Biological Cybernetics, Tübingen, Germany; i Department Pharmacy and Biochemistry, Faculty of Science, Eberhard Karls Universität Tübingen, Tübingen, Germany

**Keywords:** Quantitative MRI, Obesity, DTI, Multi-parametric mapping

## Abstract

Obesity-related structural brain alterations point to a consistent reduction in gray matter with increasing body mass index (BMI) but changes in white matter have proven to be more complex and less conclusive. Hence, more recently diffusion tensor imaging (DTI) has been employed to investigate microstructural changes in white matter structure. Altogether, these studies have mostly shown a loss of white matter integrity with obesity-related factors in several brain regions. However, the variety of these obesity-related factors, including inflammation and dyslipidemia, resulted in competing influences on the DTI indices. To increase the specificity of DTI results, we explored specific brain tissue properties by combining DTI with quantitative multi-parameter mapping in lean, overweight and obese young adults. By means of multi-parameter mapping, white matter structures showed differences in MRI parameters consistent with reduced myelin, increased water and altered iron content with increasing BMI in the superior longitudinal fasciculus, anterior thalamic radiation, internal capsule and corpus callosum. BMI-related changes in DTI parameters revealed mainly alterations in mean and axial diffusivity with increasing BMI in the corticospinal tract, anterior thalamic radiation and superior longitudinal fasciculus. These alterations, including mainly fiber tracts linking limbic structures with prefrontal regions, could potentially promote accelerated aging in obese individuals leading to an increased risk for cognitive decline.

## Highlights

• We combined DTI with quantitative multi-parametric mapping (qMPM).
• Obesity was associated with a reduction in myelin displayed by decreased R1.
• Obesity was associated with increased water content (decreased R1 and increased PD*).
• DTI revealed mainly alterations in mean and axial diffusivity with obesity.

## Introduction

Besides the strong association with numerous health conditions and changes in blood pressure, inflammation, dyslipidemia and insulin resistance (Bastard et al., 2006, Johnson et al., 2012), obesity is also related to changes in cognitive functions. These alterations include an increased risk for dementia and an accelerated cognitive decline in older age, with complementary structural and functional brain changes (Gunstad et al., 2007, Gustafson et al., 2003). Hence, there has been an increased interest in investigating obesity-related structural brain alterations using magnetic resonance imaging (MRI).

Recent studies in this fast growing field have mainly focused on changes in volume or density of gray (GM) and white matter (WM) using voxel-based (VBM) and tensor-based morphometry (TBM). A widespread reduction in GM volume and density was distinctly shown with increased BMI throughout the brain (Bobb et al., 2014, Driscoll et al., 2012, Gustafson et al., 2004, He et al., 2015, Raji et al., 2010, Taki et al., 2008, Walther et al., 2010, Ward et al., 2005, Yokum et al., 2012). Moreover, cortical thickness, a more specific and direct measure of gray matter, recently confirmed these obesity-related GM alterations showing cortical thinning with increased BMI mainly in frontal, temporal and parietal regions (Hassenstab et al., 2012, Marques-Iturria et al., 2013, Veit et al., 2014).

Macrostructural WM changes in obesity showed a more complex and less consistent pattern, revealing a positive association between BMI and WM volume/density in frontal, temporal and parietal lobes (Schwartz et al., 2014, Walther et al., 2010) and a negative relationship in the basal ganglia and corona radiata (Raji et al., 2010, Yokum et al., 2012). To clarify these findings, the microstructural composition and architecture of the white matter have been investigated using diffusion tensor imaging (DTI), which is highly sensitive to changes at the cellular and microstructural level (Alexander et al., 2007), quantifying and mapping the rate and directionality of water movement within tissue (Basser et al., 1994). Fractional anisotropy (FA) and mean diffusivity (MD) are summary measures of WM diffusivity reflecting the coherence of fiber tracts and the average rate of water diffusion, respectively. Reduced myelin integrity results in decreased FA and increased MD. It is also possible to analyze the rate of diffusion along the individual axis of the tensor: axial diffusivity (AD) measures diffusivity along the primary axis and is associated with axonal integrity, whereas radial diffusivity (RD) measures diffusivity perpendicular to the major axis reflecting myelin integrity (Basser, 1995). Increased AD can result from heightened fiber coherence or decreased axonal branching (Budde et al., 2009), while axonal damage can lead to decreased AD. Reduced myelin integrity of the membrane or sheath can also increase RD (Song et al., 2003).

Studies using DTI have revealed a loss of white matter integrity showing an inverse association between body mass index (BMI) and FA mainly in tracts within the limbic system and those connecting the temporal and frontal lobes (Bolzenius et al., 2013, Gupta et al., 2015, Ryan and Walther, 2014, Shott et al., 2014, Xu et al., 2013, Yau et al., 2014) (for review see Kullmann et al., 2015). However, the variety of obesity-related factors, such as inflammation and dyslipidemia, result in competing influences on the DTI indices. Vascular physiological factors, such as dyslipidemia and blood pressure, were related to localized higher FA, while increased BMI and global inflammation were related to a widespread reduction in FA values (Verstynen et al., 2013).

The specificity of DTI can be complemented and improved through combination with other imaging techniques such as quantitative Multi-Parametric Mapping (MPM) (Weiskopf et al., 2013) to explore specific brain tissue properties by studying a number of key contrast parameters, namely effective proton density (PD*), magnetic transfer saturation (MT), longitudinal relaxation rate (R1) and effective transverse relaxation rate (R2*). These quantitative MRI (qMRI) measures each have differential sensitivity to underlying biological metrics. MT reflects macromolecular content, with myelin being the biggest contributor in the brain (Helms et al., 2010, Schmierer et al., 2004). Hence, demyelination would lead to a reduction in MT. R1 is sensitive to the relative contribution of myelin and water content, as well as to paramagnetic content, e.g. iron (Callaghan et al., 2015, Lutti et al., 2014, Rooney et al., 2007). Thus, a reduction in myelin or an increase in water content would lead to decreased R1 levels in the brain. It should be noted that iron decreases could also lead to a reduction in R1, but to a lesser extent (Callaghan et al., 2015, Rooney et al., 2007), particularly in white matter (Gelman et al., 2001). The R2* measure is sensitive to local dephasing agents, and has been shown to correlate with iron content in the brain (Langkammer et al., 2010). Since iron is a major co-factor for the production and maintenance of myelin, a reduction of iron in the white matter may promote demyelination. Proton density is sensitive to water content, which is increased by inflammatory processes, and has been used to estimate the macromolecular tissue volume fraction (Lerski et al., 1984, Mezer et al., 2013, Tofts, 2004). Here we refer to the effective proton density since this measure has residual T2* weighting.

In the current study, we used multi-parametric mapping, in addition to DTI, to acquire quantitative maps to investigate the effect of obesity on brain white matter microstructure in young healthy adults. Given that each acquired quantitative MRI parameter is associated with particular aspects of white matter tissue structure, we can begin to detect specific changes related to obesity. We hypothesized that increased BMI and altered lipid profiles would be associated with myelin loss, and changes in water diffusion characteristics.

## Materials and methods

### Subjects

We recruited 24 healthy lean, 12 overweight and 12 obese adult participants for this study (average BMI lean group: 22.31 ± 1.71 kg/m^2^, overweight group: 27.73 ± 1.31 kg/m^2^, obese group: 34.14 ± 4.8 kg/m^2^; age range 21 to 37 years; 23 women and 25 men). DTI data were acquired in all participants. However, out of time constraints, the multi-parametric mapping protocol was additionally acquired only on 33 out of the 48 subjects (for details of this sub-cohort, please see Table 1). Informed written consent was obtained from all subjects and the local Ethics Committee approved the protocol. All participants were students at the University of Tübingen recruited using broadcast emails.

### Study design

Prior to the experiment, all participants underwent a medical examination to confirm that they did not suffer from psychiatric, neurological or metabolic diseases. Diabetes was ruled out by a 75 g oral glucose tolerance test (OGTT). Any volunteer treated for chronic disease or taking any kind of medication other than oral contraceptives was excluded. To address psychiatric diseases, the Patient Health Questionnaire (PHQ) (Löwe et al., 2002) was used. Fasting blood samples were taken to determine individuals' lipid profile (cholesterol and triglycerides) and to exclude participants with acute infection (C-reactive protein > 10 mg/l). An overview of anthropometric and metabolic characteristics is shown in Table 1.

### Data acquisition

Experiments were conducted after an overnight fast of at least 10 h and started between 8.00 and 10.00 a.m. Participants were examined on a 3T scanner (Tim Trio; Siemens) equipped with a standard 12-channel and 32-channel radio-frequency (RF) receiver head coil and RF body transmit coil. A high-resolution T1-weighted anatomical image and diffusion weighted images were acquired using the 12-channel head coil (3D MPRAGE; magnetization-prepared rapid gradient echo: matrix size = 256 × 256, 192 slices, voxel size 1 × 1 × 1 mm, TR = 2300 ms, TE = 2.98 ms, TI = 900 ms; DTI-EPI; single-shot echo planar imaging sequence: 35 nonlinear directions, 2 averages, 70 slices, diffusion weighting of b = 1000 s/mm^2^, slice thickness of 2 mm, field of view = 196 mm^2^, TR 9700 ms, TE 95 ms, acquisition matrix = 128 × 128, voxel size 1.5 × 1.5 × 2 mm). The total scanning time for the T1-weighted anatomical image and the DTI measurement was approximately 22 min.

In 33 out of the 48 participants (BMI range 19.5 to 39.3 kg/m^2^; age range 21 to 36 years; 14 women and 19 men), we additionally applied a whole-brain MPM protocol (Weiskopf et al., 2013) based on multi-echo 3D FLASH (fast low angle shot) using the 32-channel head coil to quantitatively map longitudinal relaxation rate (R1 = 1/T1), effective proton density (PD*), effective transverse relaxation rate (R2* = 1/T2*) and magnetization transfer saturation (MT). Three anatomical datasets were acquired including T1-, PD-, and MT-weighting determined by the repetition time and flip angle (T1: 18.7 ms/20°; PD and MT: 23.7 ms/6°) and by using an off-resonance, Gaussian-shaped RF pulse 2 kHz off-resonance to achieve MT weighting. Alternating gradient echoes were obtained between 2.2 and 14.7 ms at six echo times for T1- and MT-weighted acquisitions and between 2.2 and 19.7 ms at eight echo times for PD-weighted acquisitions. Further parameters were as follows: 176 slices, field-of view 156 × 240 mm, read out bandwidth = 425 Hz/pixel, GRAPPA parallel imaging (factor 2) in the phase encoding direction and 6/8 partial Fourier acquisition in the partition direction. Additional data were acquired to correct for inhomogeneities in the RF transmit field (Lutti et al., 2012). The total scanning time for the entire MPM protocol was approximately 25 min. For more details of the MPM data acquisition protocol and parameter estimation please see (Draganski et al., 2011, Helms et al., 2008a, Helms et al., 2008b, Helms and Dechent, 2009, Weiskopf et al., 2013). We visually inspected the weighted volumes for artifacts consistent with intra-scan head motion. No subject had to be excluded.

### Data analysis

#### Voxel based morphometry

The MPRAGE images were processed and examined using the VBM8 toolbox with default parameters (http://dbm.neuro.uni-jena.de/vbm.html) implemented in the SPM8 software (Wellcome Trust Centre for Neuroimaging, UCL, London, UK; http://www.fil.ion.ucl.ac.uk./spm). Total GM, WM, cerebrospinal fluid (CSF) and total intracranial volumes were extracted. The modulated volumes were smoothed with a Gaussian kernel of 6 mm full width at half maximum (FWHM).

#### DTI data analysis

DTI datasets were analyzed within the FMRIB software library framework (http://fsl.fmrib.ox.ac.uk) using a MATLAB toolbox named “pipeline for analyzing brain diffusion images” (PANDA) (Cui et al., 2013). The raw data for each participant were corrected for head motion, using eddy current correction, by registering the diffusion weighted images to the b = 0 images with an affine transformation. A brain mask was estimated using b = 0 images without diffusion weighting. Diffusion tensor metrics were then calculated (applying *dtifit* of *FSL*) in a voxel-wise fashion within the brain mask including fractional anisotropy (FA), mean diffusivity (MD), axial diffusivity (AD), and radial diffusivity (RD). For normalization, individuals' FA images in the native space were non-linearly registered to the FA template in MNI space using the *FNIRT* command of *FSL*. The resulting warping transformations were then applied to resample the images of the diffusion metrics (FA, MD, RD, AD) into MNI space with a 2 × 2 × 2 mm spatial resolution. The normalized images were then smoothed with a Gaussian kernel of 6 mm to reduce image noise and misalignment between subjects.

#### MPM data analysis and voxel-based quantification (VBQ)

To obtain quantitative maps, the data were processed using the Voxel-Based Quantification (VBQ) toolbox implemented as a toolbox for SPM8 in Matlab (The Mathworks Inc.; Natick, MA, USA). In brief, the R2* rate was determined by linear regression of the log transformed signals of the eight PD-weighted echoes. Subsequently, the first six echoes acquired for each contrast weighting were averaged to increase the signal-to-noise ratio and co-registered to each other and to a calibration map of the RF transmit field in an effort to minimize the effect of any inter-scan motion that may have occurred. These data were then used to calculate the parameter maps of the MT saturation, R1 and the effective proton density (PD*), as previously described (Helms et al., 2008a, Helms et al., 2008b, Weiskopf et al., 2013). The R1 maps were additionally corrected for RF transmit field inhomogeneities and imperfect RF spoiling (Preibisch and Deichmann, 2009). The resulting MT maps were segmented into GM and WM probability maps using the unified segmentation approach (Ashburner and Friston, 2005) and then warped to standard MNI space using the subject-specific diffeomorphic estimates derived from Dartel (Ashburner and Friston, 2005). A combined probability weighting and Gaussian smoothing (3 mm FWHM) method was applied to minimize partial volume effects and maintain the tissue-specific (for GM and WM) quantitative parameter values while normalizing the quantitative parameter maps to MNI space as described for the VBQ method (Draganski et al., 2011).

### Second-level statistics

Voxel-wise statistical analysis was performed for all DTI parameters (FA, MD, AD and RD), quantitative parameter maps of the white matter segment obtained from VBQ (PD*, R1, R2*, and MT), and WM volumes acquired from VBM. For statistical analysis, we used a multiple linear regression model in SPM8 separately for each parameter including four regressors: gender, age, total intracranial volume (ICV) and BMI as the regressor of interest. All results were thresholded at p < 0.05 family wise error corrected (FWE) within an explicit WM mask. For further analysis with SPSS (version 20), the original values of the quantitative MPM and DTI parameters were extracted from the clusters showing a significant relationship with BMI. To estimate the effect size of the relationship between BMI and the change in quantitative MPM parameters, the linear coefficient (slope) was calculated and adjusted for age, gender and total ICV. Furthermore, partial correlation analysis with the individual's lipid profile was performed (p < 0.05, corrected for number of tests). Additionally, the estimated values of the DTI and MPM metrics in regions showing a significant relationship with BMI were tested for potential gender effects. The ICBM atlas was used to label our results (Mori et al., 2008).

## Results

### Voxel-based quantification analysis (VBQ)

**Fig. 1:**
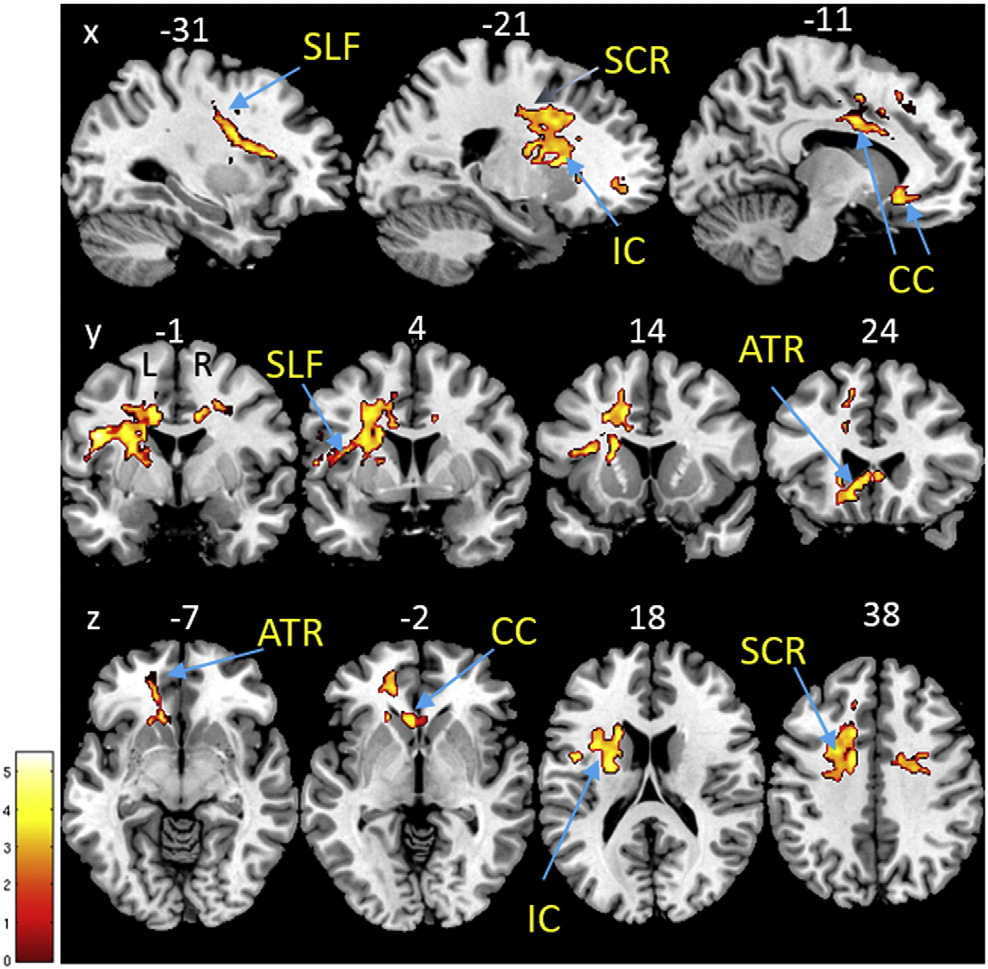
Myelin/water changes inferred from quantitative multi-parametric mapping estimated by R1 in the white matter. Statistical parameter map of regions in which R1 significantly decreased with BMI adjusted for gender, age and intracranial volume (p < 0.05, FWE-corrected). This figure is thresholded at p < 0.001 uncorrected level for display purposes only and superimposed on a T1-weighted image. The color bar indicates the t score. Abbreviations: ATR, anterior thalamic radiation; CC, corpus callosum; IC, internal capsule; R1, longitudinal relaxation rate; SCR, superior corona radiata; SLF, superior longitudinal fasciculus.

BMI was associated with significantly lower R1 within a cluster ranging from the left superior longitudinal fasciculus, anterior thalamic radiation, anterior and posterior part of the internal capsule to the body and genu of the corpus callosum (Table 2, Fig. 1) (p < 0.05, FWE-corrected). The BMI-correlated decrease in R1 amounted to 0.0062 s^− 1^ per BMI point.

PD* revealed a positive association with BMI in the right superior longitudinal fasciculus (Table 2) (p < 0.05, FWE-corrected). The BMI-correlated increase in PD* amounted to 0.167 (p.u.) per BMI point.

**Fig. 2:**
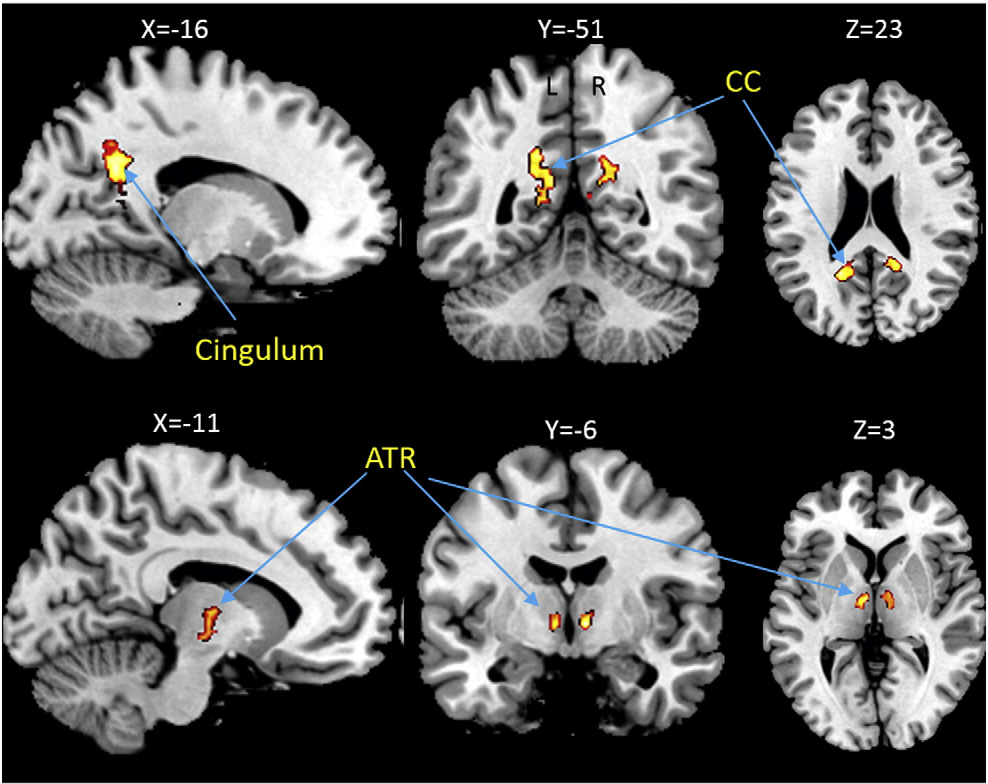
Changes in iron content inferred from quantitative maps of R2*. This statistical parameter map shows increased R2* in the cingulum and splenium of the corpus callosum and decreased R2* in the anterior thalamic radiation with BMI adjusted for gender, age and intracranial volume (p < 0.05, FWE-corrected). This figure is thresholded at p < 0.001 uncorrected level for display purposes only and superimposed on a T1-weighted image. The color bar indicates the t score. Abbreviations: ATR, anterior thalamic radiation; CC, corpus callosum; R2*, effective transverse relaxation rate.

R2* showed a negative association with BMI in the right anterior thalamic radiation and a positive association in the splenium of the corpus callosum and the cingulum (Table 2, Fig. 2) (p < 0.05, FWE-corrected). The BMI-correlated decrease in R2* ranged from 0.218 (for the corpus callosum and cingulum) to 0.286 s^− 1^ (for the anterior thalamic radiation) per BMI point.

**Fig. 3:**
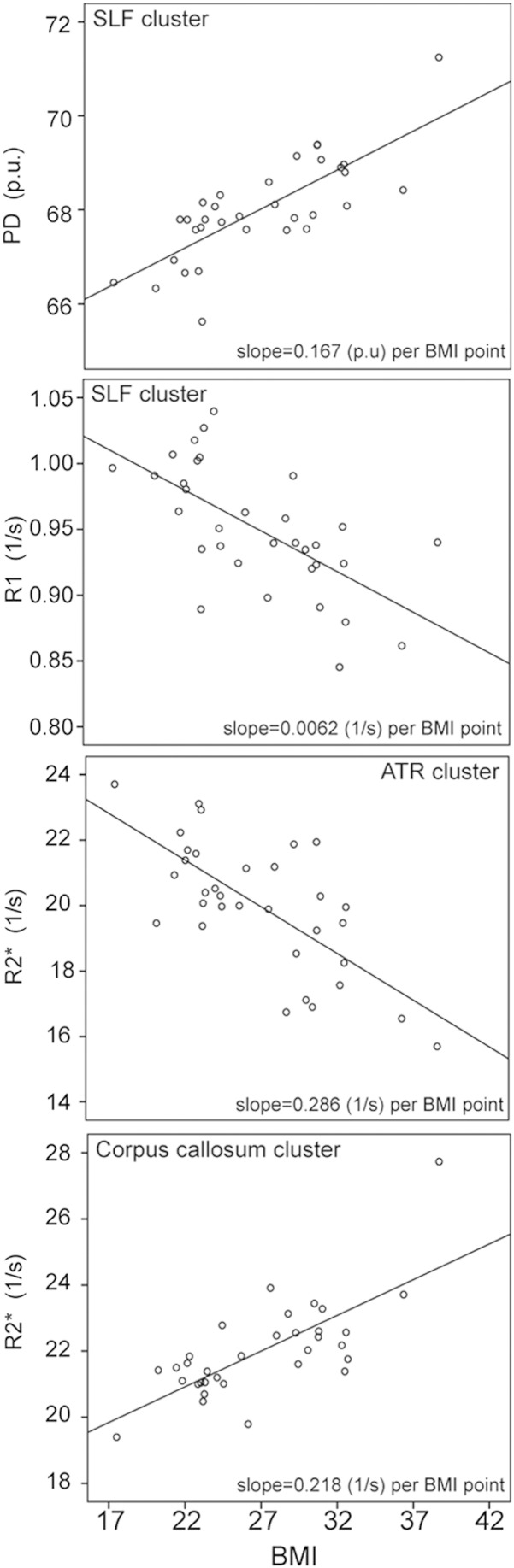
Plot depicting significant changes in quantitative multi-parametric maps of PD*, R1 and R2* in brain regions showing significant changes consistent with altered water, myelin and/or iron content with increasing BMI (kg/m^2^). These data are shown for illustrational purposes only. Abbreviations: ATR, anterior thalamic radiation; BMI, body mass index; PD*, effective proton density; R1, longitudinal relaxation rate; R2*, effective transverse relaxation rate; SLF, superior longitudinal fasciculus.

For illustration purposes, we graphically depicted the changes in PD*, R1 and R2* with increasing BMI as correlation plots in Fig. 3.

No significant correlations were identified between MT and BMI (p > 0.05, FWE-corrected).

No main effects or interactions with gender were observed for the MPM values that showed significant changes with increasing BMI.

### Diffusion tensor imaging

**Fig. 4:**
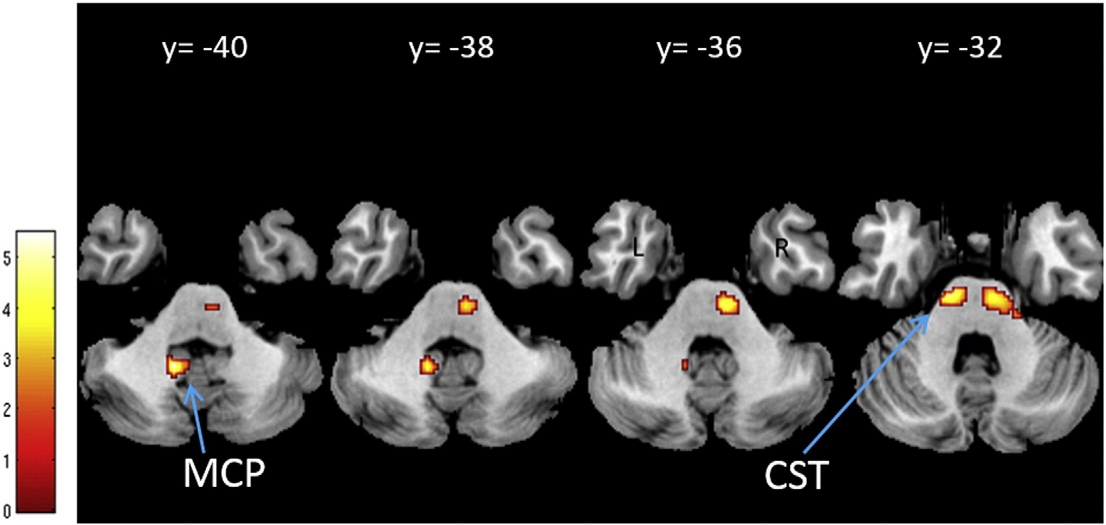
Reduced fiber integrity based on fractional anisotropy estimated by diffusion-weighted imaging. Statistical parameter map of regions in which fractional anisotropy (and radial diffusivity) significantly decreased with BMI adjusted for gender, age and intracranial volume (p < 0.05, FWE-corrected). This figure is thresholded at p < 0.001 uncorrected level for display purposes only and superimposed on a T1-weighted image. The color bar indicates the t-score. Abbreviations: CST, corticospinal tract; MCP, middle cerebellar peduncle.

**Fig. 5:**
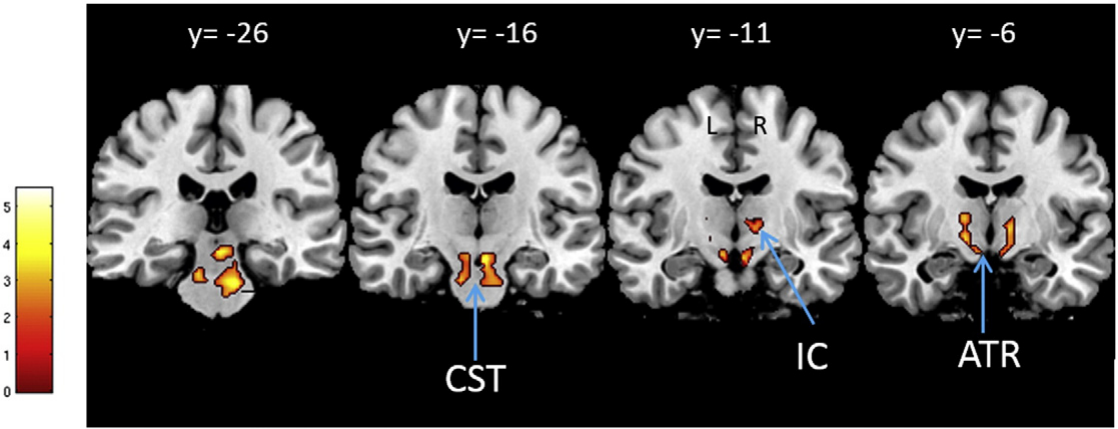
Changed white matter integrity based on mean diffusivity (MD) estimated by diffusion-weighted imaging. Statistical parameter map of regions in which MD (and also axial diffusivity) significantly decreased with BMI adjusted for gender, age and intracranial volume (p < 0.05, FWE-corrected). This figure is thresholded at p < 0.001 uncorrected level for display purposes only and superimposed on a T1-weighted image. The color bar indicates the t score. (ATR, anterior thalamic radiation; CST, corticospinal tract; IC, internal capsule).

**Fig. 6:**
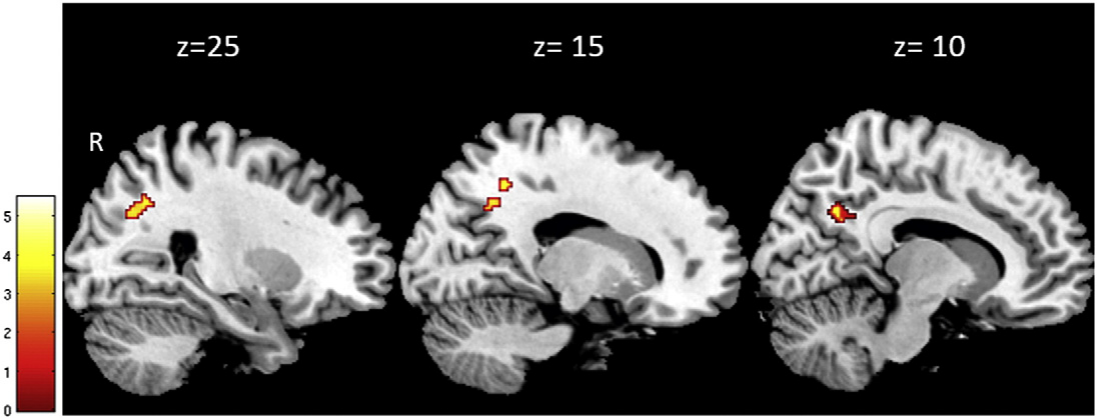
Decreased white matter integrity based on mean diffusivity (MD) estimated by diffusion-weighted imaging. Statistical parameter map of the superior longitudinal fasciculus in which MD (and also axial diffusivity) significantly increased with BMI adjusted for gender, age and intracranial volume (p < 0.05, FWE-corrected). This figure is thresholded at p < 0.001 uncorrected level for display purposes only and superimposed on a T1-weighted image. The color bar indicates the t score.

BMI was negatively associated with: FA and RD in the right middle cerebellar peduncle (Fig. 4); and MD and AD in the bilateral corticospinal tract and anterior thalamic radiation (Fig. 5). BMI was positively associated with MD and AD in the right superior longitudinal fasciculus (p < 0.05, FWE-corrected) (Table 2, Fig. 6). No main effects or interactions with gender were observed for the DTI values showing significant changes with increasing BMI.

Using VBM, we found no significant change in WM volume with increasing BMI (p > 0.05, FWE-corrected).

### Correlation analysis with lipid profiles

We performed additional correlation analysis with the clusters showing a significant relationship with increasing BMI (as reported in Table 2) and individual's lipid profile (total cholesterol, low density lipoprotein (LDL) and high density lipoprotein (HDL) cholesterol, triglycerides). The R1 cluster and the R2* of the anterior thalamic radiation cluster showed a significant positive correlation with HDL cholesterol adjusted for age, gender and ICV (r_adj_ = 0.611; p < 0.0001; r_adj_ = 0.536, p = 0.002, respectively). No significant correlation was observed with LDL cholesterol and triglycerides (p > 0.0015, corrected for number of tests).

## Discussion

We explored specific tissue properties of white matter microstructure by combining DTI with quantitative multi-parametric mapping in lean, overweight and obese young adults. Since each parameter map reflects different aspects of white matter tissue micro-architecture, specific obesity-related differences could be detected. By means of voxel-based quantification, PD*, R1, R2* and MT maps were investigated, which are particularly sensitive to water, iron and myelin content. Conventional DTI parameters included fractional anisotropy (FA), mean diffusivity (MD), axial and radial diffusivities (AD and RD).

Voxel-based quantification analysis indicated white matter parameter changes consistent with a reduction in myelin (decreased R1), increased water content (decreased R1 and increased PD*) and altered iron content (as reflected in R2*) with increasing BMI, predominantly in the superior longitudinal fasciculus (SLF), anterior thalamic radiation (ATR), internal capsule and corpus callosum.

Our results on BMI-related changes in DTI parameters revealed alterations in MD and AD mainly in the ATR, corticospinal tract and SLF. Reduced FA was mainly observed in the cerebellar peduncle. The most significant BMI-related changes in DTI parameters were in MD and AD, with a regionally specific decrease or increase in MD. Together with the strong decrease in AD, axonal damage or cellular loss could also be a possible explanation for these changes.

Using quantitative multi-parametric mapping, we identified the most extensive BMI-related differences in R1 resulting in a mean decrease of 0.006 s^− 1^ per BMI point. These differences, seen in the left SLF, ATR, internal capsule and corpus callosum, may have a number of sources, most notably demyelination, increased water content and, to a lesser extent, reduced iron content. The multi-parametric approach adopted here allows us to further unravel these effects. Although no significant effect of BMI was observed in MT, which reflects macromolecular content, i.e. typically myelin, it may be that the factors affecting MT and R2* occur concurrently and combine additively in the R1 map, making it potentially more sensitive to the particular changes occurring here (Callaghan et al., 2015). However, it is also the case that the MT maps offer a lower signal-to-noise ratio than R1 maps (Weiskopf et al., 2013).

A localized increase in the PD* maps was identified in the right SLF which could be related to increased water and/or reduced macro-molecular tissue volume fraction (Mezer et al., 2013). Moreover, MD, which is also known to increase with tissue water, showed an increase exclusively in the right SLF. Together, these measures of increased PD* and water diffusivity point to a regionally specific obesity-mediated effect in the SLF.

Iron is a major co-factor in the production and maintenance of myelin (Todorich et al., 2009). In gray matter, iron accumulates with age (Callaghan et al., 2014), while there is a reduction of iron with normal aging in white matter promoting demyelination (Callaghan et al., 2014, Draganski et al., 2011). We identified alterations in iron content (R2*) in similar areas as the observed decrease in myelin (R1) consistent with a pattern of demyelination.

Though the underlying causes are still elusive, our findings point to an important role of brain water content, which is known to be mediated by obesity-associated inflammation (Verstynen et al., 2013). Further studies are needed to investigate the influence of brain inflammatory markers on the white matter microstructure of the brain and to disentangle these effects from those of demyelination.

In the current study, BMI-related changes in R1 significantly correlated with HDL cholesterol such that higher HDL was associated with higher R1 values in the ATR, internal capsule, SLF, and in the body and genu of the corpus callosum. Similarly, Warstadt et al. (2014) found, in a sample of 403 young adults, HDL cholesterol to correlate positively with fractional anisotropy in the corpus callosum, fornix and internal capsule. Furthermore, those regions are especially vulnerable to dyslipidemia (Verstynen et al., 2013). Cholesterol is essential for myelin and synaptic formation, synaptic plasticity and receptor function. Due to the low permeability of cholesterol through the blood brain barrier, CNS cholesterol is mostly independent of serum cholesterol (Saher and Simons, 2010). Nonetheless, oxidative processes allow the conversion of cholesterol to oxysterols, which can diffuse into the brain by concentration gradients linking serum cholesterol to brain structure. Low levels of HDL cholesterol are considered a major risk factor for vascular health and are linked to verbal memory deficits in middle-aged adults (Kontush and Chapman, 2008, Singh-Manoux et al., 2008). Abnormalities in brain lipid metabolism are related to the pathogenesis of Alzheimer disease and type 2 diabetes suggesting a joint trajectory between the two diseases (Martins et al., 2006).

Anatomically, our findings of reduced WM integrity predominantly affected the corpus callosum, internal capsule, cingulum, corona radiata, SLF and the ATR. These structures mainly link limbic regions with the prefrontal cortex thereby converging reward and cognitive processes. Previous studies have found reduced white matter integrity in these structures, mainly indicated by decreased FA, in obese adolescents as well as older adults (He et al., 2015, Karlsson et al., 2013, Kullmann et al., 2015, Mueller et al., 2011, Shott et al., 2014, Stanek et al., 2011, Xu et al., 2013, Yau et al., 2014). In this current study, the ATR showed the most consistent spatial differences in DTI and multi-parametric mapping parameters, and most likely include alteration of the medial forebrain bundle (MFB) due to their close proximity (Coenen et al., 2012). These two structures converge in the anterior limb of the internal capsule and the medial prefrontal cortex, linking key limbic structures in the brain, mediating reward-seeking and appetitive motivation to maintain emotional homeostasis (Coenen et al., 2012, Mori et al., 2008). The BMI-associated white matter alterations of PD*, R1, R2* and fractional anisotropy also include parts of the ATR/MFB subcortical projection pathways. The superior longitudinal fasciculus showed widespread alterations with increased BMI in water diffusivity, R1 and PD*. The SLF is a major intra-hemispheric fiber tract connecting the posterior part of the brain with the prefrontal cortex. Different measures of white matter microstructure affected separate components of the SLF. Axial and mean diffusivity increased with BMI in the occipital part, while R1 decreased with BMI in the frontal operculum and increased PD* was observed in the temporal part. All SLF components terminate in the dorsolateral prefrontal cortex, a prominent brain region highly relevant for decision making processes and executive function (Makris et al., 2005). Moreover, recent anatomical as well as functional connectivity studies revealed an obesity-associated imbalance between reward and executive brain networks. A dissociable pattern of structural connectivity was observed, using deterministic tractography, showing increased fiber tract density between reward network regions and decreased fiber tract density between prefrontal regions (Gupta et al., 2015). In addition, functional connectivity brain networks of the limbic and prefrontal system are especially vulnerable to increased body weight (Garcia-Garcia et al., 2013, Kullmann et al., 2012, Kullmann et al., 2013, Marques-Iturria et al., 2015). The prominent decrease in WM integrity of the MFB/ATR and SLF in obese adults could contribute to over-eating via the rich projections to and from the prefrontal cortex.

The primary limitation of the current study is the small sample size. Hence, interpretations on possible interactions between BMI and gender are difficult. Furthermore, our sample is relatively young not reflecting the population at large. Developmental aspects of the younger subjects could also play an important role, as increased body weight and fat impact the brains microstructure differently. Ou et al. (2015) recently showed increased FA in obese children, the opposite of what has been found in obese adults. Limitations shared by all morphometric and VBQ studies are potential registration and segmentation errors, as well as partial volume effects. These sources of bias have been minimized in this study by using the Dartel algorithm for inter-subject registration, which has been shown to result in maximally accurate registration (Klein et al., 2009), and by using a specific normalization procedure on the multi-parameter maps to preserve the correct quantitative values (Draganski et al., 2011). Furthermore, while we measured CRP to exclude acute inflammation, obesity is associated with low grade inflammation, which is known to have a widespread and complex impact on brain microstructure, prohibiting a unique interpretation of the observed parameter changes (see Weiskopf et al., 2015 for a discussion of the sensitivity and specificity of qMRI measures).

In summary, we identified specific white matter microstructure changes related to obesity in healthy young adults. Predominantly R1, R2*, MD and AD showed local changes with increased BMI in white matter fiber tracts linking key limbic structures with prefrontal regions, possibly explaining the increased risk for cognitive impairments and dementia in obesity in older age. R1 seemed to be particularly sensitive to increased BMI, indicative of a loss of white matter integrity either via demyelination or inflammatory effects. Hence, changes in quantitative multi-parametric mapping parameters could be possible precursors to a loss in white matter integrity in obese individuals.

## Figures and Tables

**Table 1:** Participants' characteristics (n = 33).

	Lean	Overweight	Obese	p
Gender (female/male)	6/10	3/5	5/4	0.669
Age (y)	26.68 ± 3.68	26.12 ± 1.95	26.88 ± 4.45	0.902
Body mass index (kg/m^2^)	22.43 ± 1.61	28.13 ± 1.38	33.16 ± 3.16	< 0.001
Cholesterol (mg/dl)	174.93 ± 28.39	169.37 ± 19.92	172.33 ± 30.26	0.893
HDL cholesterol (mg/dl)	62.56 ± 4.15	49.75 ± 3.46	43.00 ± 2.90	0.002
LDL cholesterol (mg/dl)	85.37 ± 20.48	89.62 ± 12.79	92.33 ± 28.17	0.726
Triglyceride (mg/dl)	76.37 ± 20.71	92.00 ± 61.79	134.11 ± 78.66	0.047
C-reactive protein (mg/l)	0.12 ± 0.17	0.17 ± 0.15	0.50 ± 0.44	0.004
Ferritin (μg/dl)	4.83 ± 4.04	5.78 ± 4.83	8.14 ± 8.86	0.851

Data are presented as mean ± SD. p = p-Values for comparison of unadjusted log*_e_* transformed data by ANOVA.

**Table 2:** BMI-related white matter changes.

Regions	Hem	Cluster size	MNI (mm) (x,y,z)	T value	p_FWE − corr_
*DTI parameters (n = 48; 25 lean and 23 obese participants)*					
FA maps: negative correlation with BMI					
Middle cerebellar peduncle	R	19	19,− 52,− 40	5.86	0.014*
MD maps: negative correlation with BMI					
Corticospinal tract	R/L	91s7	± 10,− 22,− 26	5.49	0.0001
Anterior thalamic radiation	R/L		± 10,− 6,− 6	5.22	
Anterior limb of internal capsule	L		− 12,2,− 2	4.64	
Posterior limb of internal capsule	R/L		± 10,− 8,0	4.21	
MD maps: positive correlation with BMI					
Sup longitudinal fasciculus	R	330	42,− 56,12	5.13	0.005
AD maps: negative correlation with BMI					
Corticospinal tract	R/L	1113	± 10,− 22,− 24	6.38	< 0.001
Anterior thalamic radiation	R/L		± 14,− 6,− 4	5.41	
Anterior limb of internal capsule	L		− 10,2,− 2	4.21	
Posterior limb of internal capsule	R		14,− 6,− 4	4.05	
AD maps: positive correlation with BMI					
Sup longitudinal fasciculus	R	323	42,− 68,26	6.40	0.007
RD maps: negative correlation with BMI					
Middle cerebellar peduncle	R/L	180	± 12,− 20,− 28	5.13	0.006

*Voxel-based-quantification results (n = 33; 16 lean and 17 obese participants)*					
PD* map: positive correlation with BMI					
Superior longitudinal fasciculus	R	62	52,− 37,− 6	6.26	0.049*
R1 map: negative correlation with BMI					
Superior longitudinal fasciculus	L	4831	− 45,− 1,24	5.26	< 0.001
Anterior thalamic radiation/anterior limb of internal capsule			− 22,11,10	4.80	
Body of corpus callosum			− 12,0,34	4.72	
Posterior limb of internal capsule			− 24,− 7,18	4.67	
Cingulum			− 12,15,37	4.62	
Anterior thalamic radiation			− 18,23,− 2	4.49	
Anterior limb of internal capsule			− 24,2,16	4.48	
Genu of corpus callosum			− 6,20,− 2	4.43	
Superior corona radiata			− 27,2,24	4.38	
Superior longitudinal fasciculus			− 33,8,18	4.37	
R2* map: negative correlation with BMI					
Anterior thalamic radiation	R/L	189	± 8,− 6,− 2	5.06	0.022
R2* map: positive correlation with BMI					
Splenium of the corpus callosum	L	494	− 18,− 52,24	4.88	< 0.001
Cingulum			− 15,− 52,31		
Splenium of the corpus callosum	R	250	15,− 46,25	4.78	0.011
Cingulum			10,− 45,16		

Data were analyzed using multiple regression analyses in SPM8; correlations with BMI were adjusted for gender, age and total intracranial volume. Results survived a whole-brain cluster level threshold corrected for multiple comparisons of p_FWE_ < 0.05; *p_FWE_ < 0.05 peak-level. Abbreviations: AD, axial diffusivity; DTI, diffusion tensor imaging; FA, fractional anisotropy; MD, mean diffusivity; PD*, effective proton density; RD, radial diffusivity; R1, longitudinal relaxation; R2*, effective transverse relaxation.
